# Inequalities in Access to Specialist Allergy Services in the United Kingdom: A Report From the BSACI Registry for Immunotherapy (BRIT)

**DOI:** 10.1111/cea.70034

**Published:** 2025-04-01

**Authors:** Mich Erlewyn‐Lajeunesse, Luciano Perfetti Villa, Shifa Shaikh, Maria Smith, Vasiliki Balodima, Sarah Baker, Tom Dawson, Pamela Ewan, Sujoy Khan, Deborah Marriage, Louise Michaelis, Leyla Pur Ozygit, Anna Thursby‐Pelham, Amena Warner, Olga Maslovskaya, the BRIT Practitioners Group, Hana Alachkar, Saul Berkovitz, Malini Bhole, Thippeswamy Billahallli, Helen A Brough, Natalia Cartledge, José Maia e Costa, Beverley Fish, Minal Gandhi, Pavels Gordins, Elizabeth Griffiths, Sophie Hadfield, Haseena Jearally, Anjli Jethwa, Leah Jones, Sonia Lal, Claire Leck, Sian Ludman, Susana Marinho, Joanne McCullough, Anne McDonnell, Hayley Meers, Phoebe Moulsdale, Lizanne Noronha, Nandinee Patel, Julie Pentland, Raul Scott Pereira, Graham Roberts, Jennifer Rowley, Anna Rucker, Michael Rudenko, Chris Rutkowski, Penny Salt, Aida Semic‐Jusufagic, Punit Shah, Sarah Sholtysek, Adrian Sie, James Stewart, Gary Stiefel, Iason Thomas, David Tuthill, Gillian Vance, Shaun Walter, Patrick Yong, Natasha Zurick

**Affiliations:** ^1^ University of Southampton and Southampton Children's Hospital Southampton UK; ^2^ Department of Social Statistics and Demography University of Southampton Southampton UK; ^3^ British Society for Allergy and Clinical Immunology London UK; ^4^ University Hospital Southampton Southampton UK; ^5^ Anaphylaxis UK Farnborough UK; ^6^ Worcestershire Acute Hospitals NHS Trust Worcester UK; ^7^ Cambridge University Hospitals NHS Foundation Trust and University of Cambridge Clinical School Cambridge UK; ^8^ Castle Hill Hospital Cottingham UK; ^9^ University Hospitals Bristol and Weston Bristol UK; ^10^ Great Northern Children's Hospital UK; ^11^ Glenfield Hospital University Hospitals of Leicester NHS Trust Leicester UK; ^12^ University Hospital Bristol and Weston NHS Trust Bristol UK; ^13^ Allergy UK UK

**Keywords:** allergen, allergy, basic immunology, biologics, clinical immunology, food allergy, immunotherapy, immunotherapy and tolerance induction, venom and insect allergy

## Abstract

**Background:**

There is an unmet need for specialist allergy treatment in the United Kingdom. Allergen immunotherapy and treatment with omalizumab for chronic spontaneous urticaria (CSU) are key markers for these services. The British Society for Allergy and Clinical Immunology (BSACI) Registry for Immunotherapy (BRIT) is a national project to record the real‐world effectiveness, safety and access to treatment for aero‐allergen, venom and peanut immunotherapy as well as omalizumab for CSU.

**Methods:**

We described participant demographics, the index of multiple deprivation (IMD) and access to treatment from the registry launch. Data for 1835 participants were available for analysis from 63 centres enrolled between 1st October 2018 and 24th August 2023.

**Results:**

96.5% (1771/1835) were living in England, with only 3.5% (64) being from the devolved nations. 14.4% (251/1748) were in the most affluent IMD decile compared to 4.5% (78/1748) in the most deprived IMD decile. White participants were 1.74 times more likely to be referred directly from primary care compared to people of Asian, black, mixed or other minority ethnic groups. Instead, these groups were referred more frequently from secondary or tertiary hospital services. The median distance travelled from home to the treatment centre was 15.2 miles, with evidence of clustering around specialist centres.

**Conclusions:**

We have described disparities and unwarranted variation in the provision of treatment around the UK. The data suggest that there is limited access to immunotherapy in the devolved nations. Access is also reduced by socioeconomic deprivation. White participants were more likely to receive a direct referral from primary care than those from other ethnic groups whose referral pathways were more complex. Registry data are limited by participant enrolment and may have selection bias. Nevertheless, BRIT has highlighted inequity in access to specialist allergy services in the UK.

## Graphical abstract

The BRIT registry highlights disparities in access to allergy treatments in the UK, with socio‐economic and ethnic factors affecting care availability.

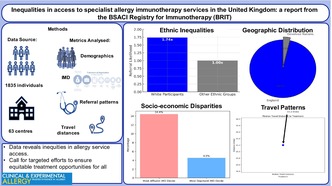

**Summary**

- There may be geographical inequality in access to specialist allergy services and biologics
- Results suggest reduced access to specialist care for the most deprived and minority ethnic groups
- Registry data are limited by those who consent to participate and may have selection bias

## 1 Introduction

For decades there has been a desire to improve coverage in allergy services [1]. Allergists are trained to manage a variety of diseases that are also the remit of other organ‐based specialists. Specific allergen immunotherapy is one area of practice that is unique to allergy medicine, where allergists would be expected to lead practice and develop guidelines. Immunotherapy is much less accessible in the United Kingdom than in other European countries and despite state‐funded services being free at the point of care [2]. In continental Europe, allergists are mainly office‐based specialists practising within a primary care environment, while in the UK, specialists are limited to hospital‐based centres. Several treatments are used by allergy specialists in the UK; these include aero‐allergen Immunotherapy (AIT) to common environmental allergens such as grass pollen and house dust mite, and venom immunotherapy (VIT) for those who have experienced a systemic allergic reaction to insect stings [3, 4]. Allergists also prescribe high‐cost drugs for biological immune modulation, such as the use of anti‐immunoglobulin E (omalizumab, OMA) for the treatment of chronic spontaneous urticaria (CSU) and asthma [5]. Peanut oral immunotherapy (PIT) has also recently been licensed and approved for prescription through publicly funded services [6]. Mapping specific allergen immunotherapy practice and the use of high‐tariff immunomodulation in the UK could be a useful marker for allergy services as a whole and help to define the ‘unmet need’ and unwarranted variation [7].

It is important to note that the exact number of centres offering paediatric and adult immunotherapy is not known. Other groups have looked at the provision of allergen immunotherapy and biologicals in the UK. Most recently, a nationwide survey of paediatric allergy services showed that 60 centres offered AIT, 18 VIT and 25 OMA, and most had small numbers of patients [7]. Over a decade ago, Rajakulasingham and colleagues surveyed 22 adult allergy centres in the UK and reviewed the care of 1731 allergen immunotherapy patients [8]. They found that half of these centres were led by consultants who did not practise allergy as their main specialty. Vance et al. surveyed practice with results from 12 paediatric centres and data from over 300 children receiving allergen immunotherapy [9]. Both surveys described the piecemeal provision of allergen immunotherapy in line with estimates suggesting that the UK lags behind other European countries in the delivery of allergen immunotherapy [10]. The use of Omalizumab for the treatment of CSU has been described in multicentre surveys and as part of the prospective international AWARE collaboration and the international CURE registry [11, 12, 13, 14]. None of these surveys addressed access to therapy, reporting instead on formulation, route of administration and safety in clinical practice. Previous surveys of VIT in the UK have had good responses, with up to 95% of surveyed centres contributing to a questionnaire on clinical practice [15, 16]. About half of surveyed centres had fewer than ten or no patients under current treatment. With multiple small throughput centres practising independently, a national registry can help to benchmark services against national trends, improve adherence to clinical guidelines and best practice and provide a more comprehensive picture across the UK when all treatments are registered in one place.

We developed a participant registry to record the access to treatment, safety and real‐world effectiveness of allergen immunotherapy and high‐cost immunomodulatory treatment by allergists in the UK. A registry allows the collection of data about patients that have a common disease or have received certain treatments across multiple sites [17]. Web‐based patient registries are a well‐tested tool for collecting data on the real‐world use of specialist treatments [18]. They have been used successfully across a wide variety of clinical settings [19]. They are increasingly recognised as an important source of valuable real‐world evidence to inform practice. A recent review placed their contribution second only to randomised clinical trials [20].

The BSACI Registry for Immunotherapy (BRIT) was launched 5 years ago and has grown steadily over time. The registry records episodes of treatment (rather than individual appointments) and allows the participants themselves to enter effectiveness and quality of life data, as well as adverse reactions, particularly those that lead to treatment discontinuation. In this article, we describe the geographic, demographic and some socioeconomic factors that are associated with access to specialist immunotherapy care.

## 2 Methods

BRIT is a web‐based registry that can be accessed securely using a standard web browser.

Its Use Is Voluntary and Is Available Free of Charge to all Consultant Members of BSACI. It Is Open to Participants Who Are Resident in the UK. The Registry Allows Clinicians to Enter Participant Information Onto a database, whether in hospital or From an Office‐Based Practice. It Collects Data for Both NHS and Private Practice Users. BRIT Is a Research Database Approved by the West of Scotland Research Ethics Committee 4 (IRAS Number 249481). The Data Used for the Analysis Were Collected Between 1st October 2018 and 24th August 2023.

### 2.1 Participant Enrolment

Patients are invited to join the registry by their supervising consultant or their delegated users, such as clinical nurse specialists, clinical administrators and doctors of both consultant and nonconsultant grade working in the immunotherapy clinic. There is a range of participant information leaflets available for adults, children and their parents. Participants must provide written informed consent to join the registry, which is then kept in local medical notes. The registry collects personally identifiable information including their home postcode and self‐identification of gender and ethnic group with the specific permission of the participant. Ethnicity categories were mapped to those of the 2011 National Census (Ethnicity and National Identity in England and Wales‐Office for National Statistics) (ons.gov.uk). Participants are contacted by the registry at intervals to record safety and effectiveness data and monitor their own care through the completion of online patient‐related outcome measures (PROM).

### 2.2 Statistical Analysis

In this paper, we describe how patient demographics, socioeconomic factors, ethnicity and distance travelled are factors in accessing the real‐world use of immunotherapy. We employed descriptive statistical techniques for the analysis. No inferential statistics are produced as ideally the registry should contain full coverage of all treatment episodes across the UK during the given period and therefore, there should be no need to generalise findings to the wider population of the patients in the UK. Additionally, the registry data are not collected using a probability‐based sampling approach and therefore, it is sufficient to produce relevant descriptive statistics and comment on missing data points.

## 3 Results

Data for this analysis were provided by the clinical teams supporting 96 consultants from 63 sites across the United Kingdom (UK). Our analytical sample contained 1835 participants for whom full information was available, all of whom were receiving various forms of immunotherapy or immunomodulating treatment. The demographic characteristics of registry participants are shown in Table 1. Children made up 45.0% (832/1835) of participants. Most patients were living in England (96.5% (1771/1835)), with 65% of the sample living outside London and 31.5% living within Greater London. The devolved nations were poorly represented, with 0.4% of participants from Scotland, 1.7% from Wales and 1.4% from Northern Ireland. In our sample, 1296 (71.0%) received allergen immunotherapy, 318 (17.0%) venom immunotherapy, 172 (9.0%) Omalizumab for the management of chronic spontaneous urticaria, and 49 (3.0%) peanut oral immunotherapy. There were only two venom participants recorded in Wales, with no registered participants otherwise for VIT, OMA, or PIT in the devolved nations. 97.7% were being treated by the National Health Service (NHS). Consequently, private practice made up 2.3% of immunotherapy services overall, except for peanut immunotherapy, where private episodes of care represented 30.6% (15/49) of all treatments.

**TABLE 1:** Demographics of registry participants by type of allergen immunotherapy.

Variable	Allergen immunotherapy (AIT)	Venom immunotherapy (VIT)	Omalizumab for CSU (MAB)	Peanut immunotherapy (PIT)	Total
Gender					
Male	812 (62.7%)	174 (54.7%)	36 (20.9%)	26 (53.1%)	1048 (57.1%)
Female	484 (37.3%)	144 (45.3%)	135 (78.5%)	23 (46.9%)	786 (42.8%)
Unknown	0 (0.0%)	0 (0.0%)	1 (0.6%)	0 (0.0%)	1 (0.1%)
Age groups					
0–15	737 (56.9%)	24 (7.5%)	29 (16.9%)	42 (85.7%)	832 (45.3%)
16–24	188 (14.5%)	5 (1.6%)	29 (16.9%)	7 (14.3%)	229 (12.5%)
25–34	152 (11.7%)	12 (3.8%)	32 (18.6%)	0 (0.0%)	196 (10.7%)
35–44	122 (9.4%)	32 (10.1%)	29 (16.9%)	0 (0.0%)	183 (10.0%)
45–54	65 (5.0%)	59 (18.6%)	27 (15.7%)	0 (0.0%)	151 (8.2%)
55–64	23 (1.8%)	98 (30.8%)	17 (9.9%)	0 (0.0%)	138 (7.5%)
65+	9 (0.7%)	88 (27.7%)	9 (5.2%)	0 (0.0%)	106 (5.8%)
Ethnicity					
White	981 (75.7%)	311 (97.8%)	152 (88.4%)	39 (79.6%)	1483 (80.8%)
Asian or Asian British	129 (10.0%)	2 (0.6%)	13 (7.6%)	5 (10.2%)	149 (8.1%)
Black, Black British, Caribbean or African	59 (4.6%)	1 (0.3%)	0 (0.0%)	0 (0.0%)	60 (3.3%)
Mixed or multiple ethnic groups	127 (9.8%)	4 (1.3%)	7 (4.1%)	5 (10.2%)	143 (7.8%)
Country of residence^a^					
England—Greater London	464 (35.8%)	79 (24.8%)	23 (13.4%)	12 (24.5%)	578 (31.5%)
England outside London	770 (59.4%)	237 (74.5%)	149 (86.6%)	37 (75.5%)	1193 (65.0%)
Scotland	7 (0.5%)	0 (0.0%)	0 (0.0%)	0 (0.0%)	7 (0.4%)
Wales	29 (2.2%)	2 (0.6%)	0 (0.0%)	0 (0.0%)	31 (1.7%)
Northern Ireland	26 (2.0%)	0 (0.0%)	0 (0.0%)	0 (0.0%)	26 (1.4%)
Practice					
NHS	1271 (98.1%)	318 (100.0%)	169 (98.3%)	34 (69.4%)	1792 (97.7%)
Private	25 (1.9%)	0 (0.0%)	3 (1.7%)	15 (30.6%)	43 (2.3%)
Referral source					
Self‐referral	22 (1.7%)	1 (0.3%)	4 (2.3%)	13 (26.5%)	40 (2.2%)
Primary care provider	419 (32.3%)	201 (63.2%)	54 (31.4%)	2 (4.1%)	676 (36.8%)
Secondary care provider	407 (31.4%)	42 (13.2%)	51 (29.7%)	16 (32.7%)	516 (28.1%)
Tertiary/regional care provider	354 (27.3%)	49 (15.4%)	20 (11.6%)	13 (26.5%)	436 (23.8%)
Other	94 (7.3%)	25 (7.9%)	43 (25.0%)	5 (10.2%)	167 (9.1%)
Distance from treatment centre					
Short distance (less than 9.2 miles)	485 (37.4%)	49 (15.4%)	62 (36.0%)	13 (26.5%)	609 (33.2%)
Medium distance (between 9.2 and 20 miles)	342 (26.4%)	81 (25.5%)	36 (20.9%)	19 (38.8%)	478 (26.0%)
Long distance (more than 20 miles)	469 (36.2%)	188 (59.1%)	74 (43.0%)	17 (34.7%)	748 (40.8%)
Total^b^	1296 (71%)	318 (17%)	172 (9%)	49 (3%)	1835 (100%)

*Note:* Column percentage in parentheses.

^a^ Channel Islands/Isle of Man is contained in England outside London.

^b^ Row percentage in parentheses.

### 3.1 Index of Multiple Deprivation (IMD)

**FIGURE 1:**
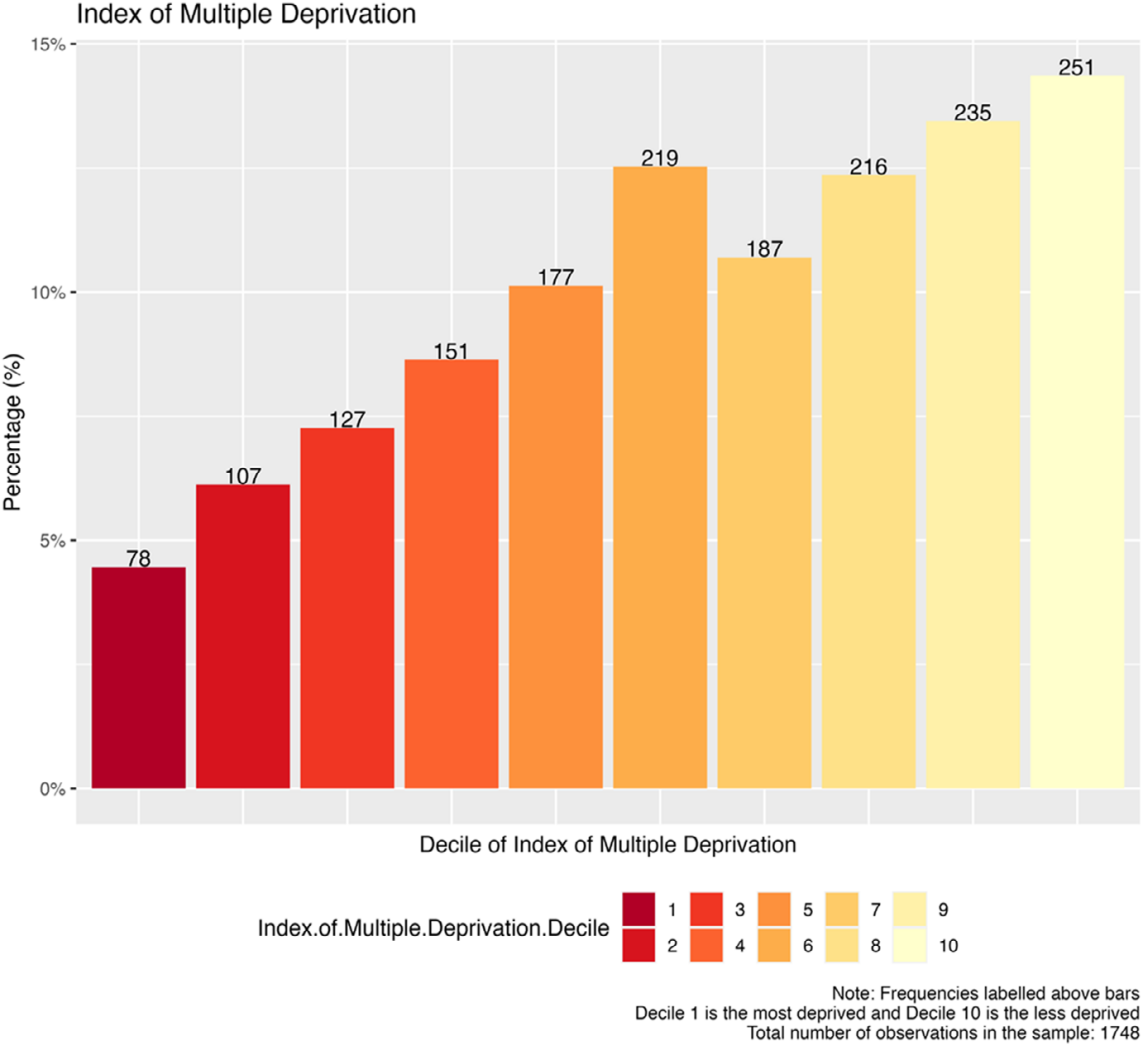
Participants stratified by index of multiple deprivation (IMD). The bar chart shows registry participants in England and Wales according to the IMD decile rank of their home postcode. The first decile is the most deprived (shaded bars) the tenth decile is the least.

**FIGURE 2:**
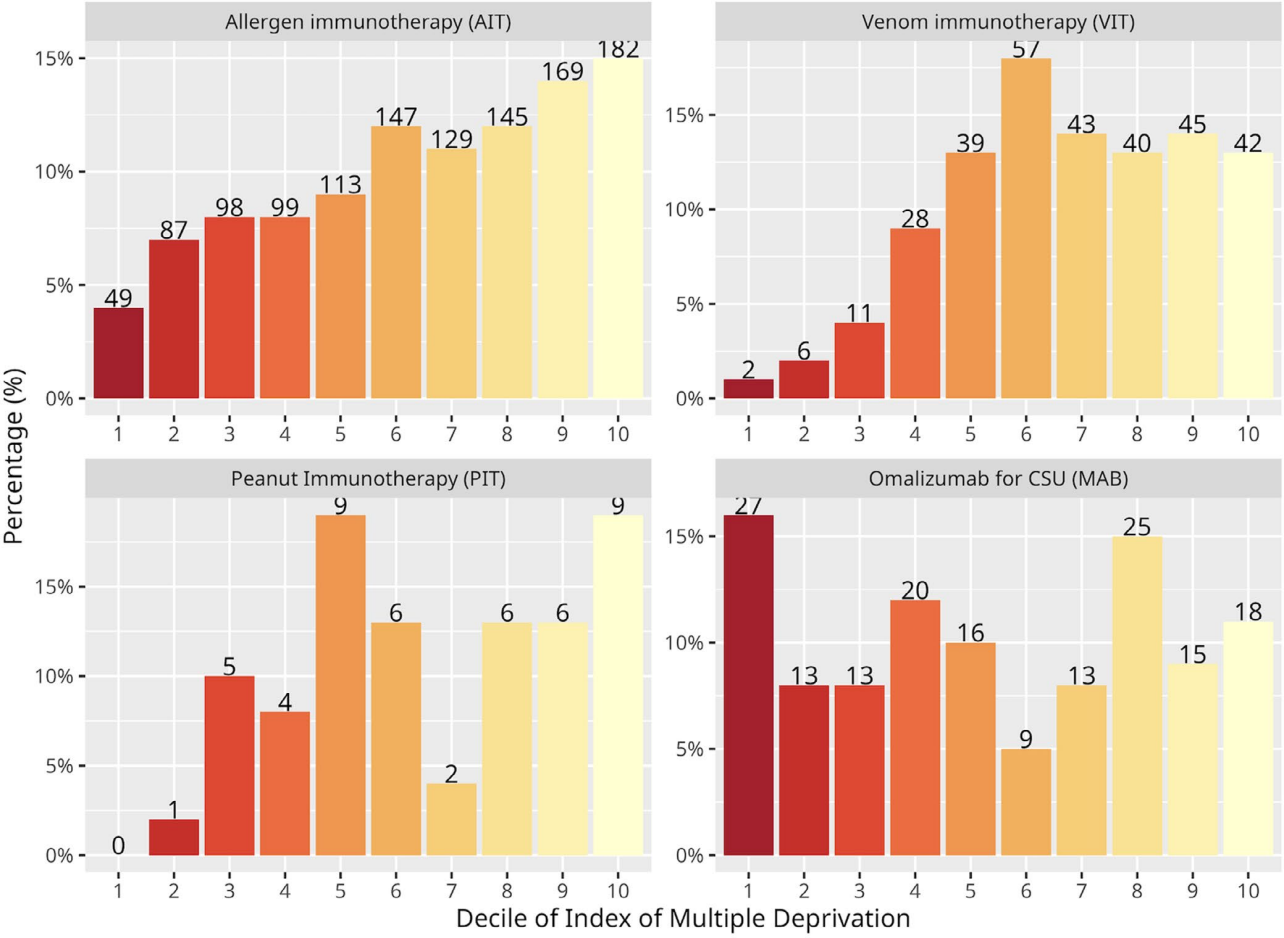
Index of multiple deprivation (IMD) by treatment domain. Where decile 1 is the most deprived and decile 10 is the most affluent.

We employed the index of multiple deprivation (IMD) based on home postcode for participants being treated in England and Wales [21]. (The IMD is not available for people living in other parts of the UK, although other tools are available but were not employed due to the small number of participants in those regions). There was a clear disparity between the proportion in the upper socioeconomic deciles compared to those in the most deprived deciles (Figure 1). In the registry, 14.4% (251/1748) were found to be in the most affluent decile compared with only 4.5% (78/1748) in the most deprived. Participants were 3.2 times more likely to receive immunotherapy in the most affluent decile than in the least. This trend was most marked for VIT at 21.0 (42/2), AIT 3.7 (182/49), and PIT 9.0 (9/1 from second decile as no participants in first) but not for OMA 0.7 (18/27) (Figure 2). The IMD is made of several component rankings including health, employment, education and skills, and income. At 4.9 (328/67) health deprivation deciles showed the most marked difference between the most and least affluent participants. Differences between the most and least affluent were also seen in the employment deciles (3.9 (277/71)). Other IMD indicators showed similar ratios to those seen overall: 3.4 (288/85) for education and skills deciles, and 3.3 (273/82) for income deciles.

### 3.2 Referral Pathways to Access Care

**FIGURE 3:**
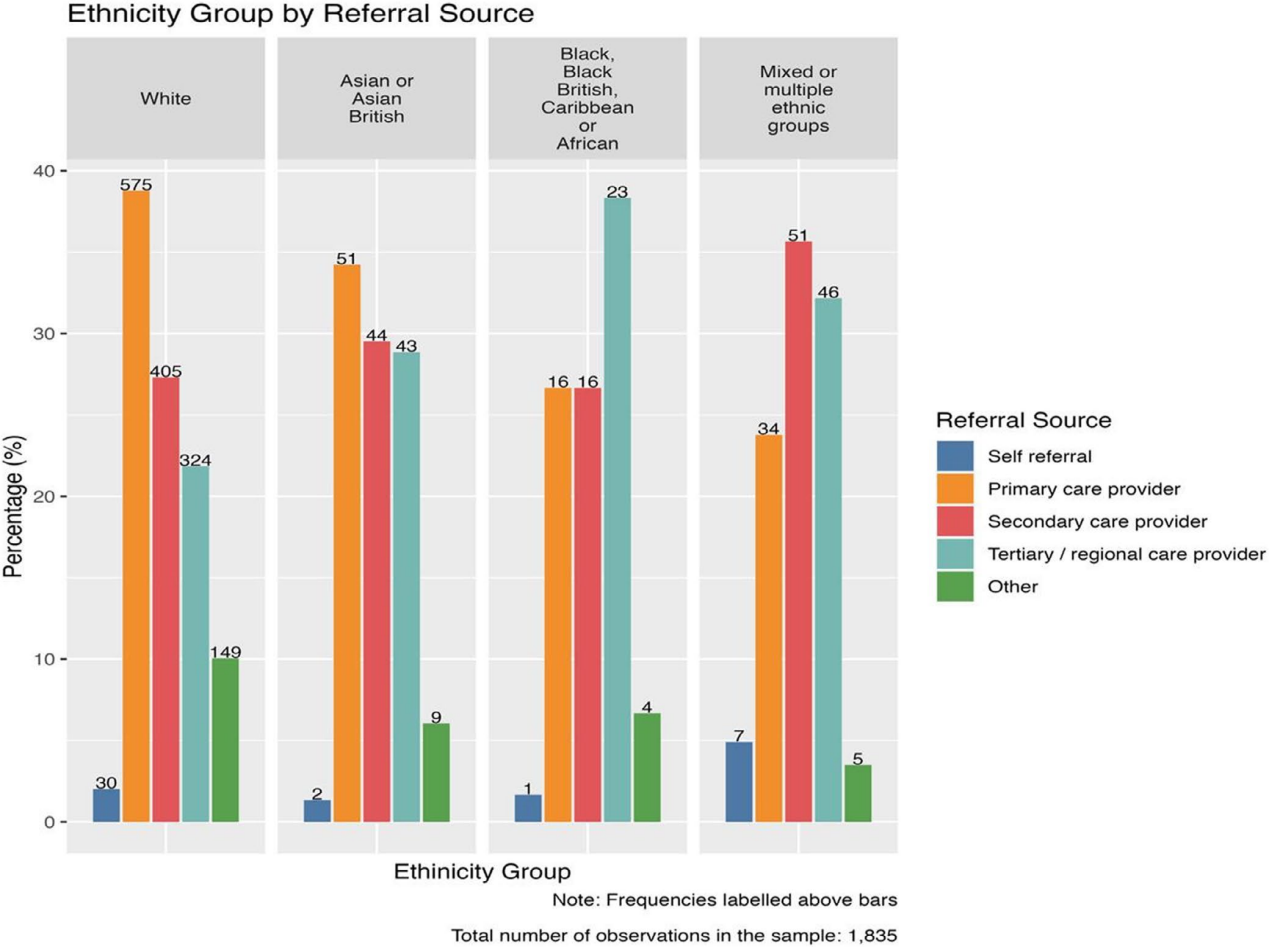
Bar chart showing referral patterns to specialist allergy services by ethnicity.

There were differences in referral patterns to specialist allergy services observed between different ethnic groups (Figure 3). All groups were more likely to be referred from a secondary or tertiary hospital service than directly from primary care (referral ratio of primary to secondary OR tertiary care of 0.71 (676/952)) (Table 2). This ratio varied between ethnic groups, and the primary care referral rate was higher for white and Asian participants. Primary care referral ratios were 0.79 for white, 0.59 for British Asian, 0.41 for black British and 0.40 for those of mixed or other ethnicities. White participants were 1.35 times more likely to be referred from primary care than British Asians, 1.92 times more likely than black British, and 2.25 times more likely than people of mixed or other ethnic groups. Overall, white participants were 1.74 times more likely to be referred directly from primary care compared with other ethnic groups.

**TABLE 2:** Participant demographics by referral source.

Name	Self‐referral	Primary care provider	Secondary care provider	Tertiary/regional care provider	Other	Total^a^
Ethnicity						
White	30 (2.0%)	575 (38.8%)	405 (27.3%)	324 (21.8%)	149 (10.0%)	1483 (80.8%)
Asian or Asian British	2 (1.3%)	51 (34.2%)	44 (29.5%)	43 (28.9%)	9 (6.0%)	149 (8.1%)
Black, Black British, Caribbean or African	1 (1.7%)	16 (26.7%)	16 (26.7%)	23 (38.3%)	4 (6.7%)	60 (3.3%)
Mixed or multiple ethnic groups	7 (4.9%)	34 (23.8%)	51 (35.7%)	46 (32.2%)	5 (3.5%)	143 (7.8%)
Country Of Residence^b^						
England—Greater London	20 (3.5%)	190 (32.9%)	186 (32.2%)	176 (30.4%)	6 (1.0%)	578 (31.5%)
England outside London	20 (1.7%)	470 (39.4%)	286 (24.0%)	260 (21.8%)	157 (13.2%)	1193 (65.0%)
Scotland	0 (0.0%)	1 (14.3%)	5 (71.4%)	0 (0.0%)	1 (14.3%)	7 (0.4%)
Wales	0 (0.0%)	10 (32.3%)	21 (67.7%)	0 (0.0%)	0 (0.0%)	31 (1.7%)
Northern Ireland	0 (0.0%)	5 (19.2%)	18 (69.2%)	0 (0.0%)	3 (11.5%)	26 (1.4%)
Type of immunotherapy						
Allergen immunotherapy (AIT)	22 (1.7%)	419 (32.3%)	407 (31.4%)	354 (27.3%)	94 (7.3%)	1296 (70.6%)
Venom immunotherapy (VIT)	1 (0.3%)	201 (63.2%)	42 (13.2%)	49 (15.4%)	25 (7.9%)	318 (17.3%)
Omalizumab for CSU (MAB)	4 (2.3%)	54 (31.4%)	51 (29.7%)	20 (11.6%)	43 (25.0%)	172 (9.4%)
Peanut immunotherapy (PIT)	13 (26.5%)	2 (4.1%)	16 (32.7%)	13 (26.5%)	5 (10.2%)	49 (2.7%)
Distance from treatment centre						
Short distance (less than 9.2 miles)	11 (1.8%)	216 (35.5%)	179 (29.4%)	137 (22.5%)	66 (10.8%)	609 (33.2%)
Medium distance (between 9.2 and 20 miles)	12 (2.5%)	194 (40.6%)	137 (28.7%)	99 (20.7%)	36 (7.5%)	478 (26.0%)
Long distance (more than 20 miles)	17 (2.3%)	266 (35.6%)	200 (26.7%)	200 (26.7%)	65 (8.7%)	748 (40.8%)
Total	40 (2%)	676 (37%)	516 (28%)	436 (24%)	167 (9%)	1835 (100%)

*Note:* Row percentage in parentheses.

^a^ Column percentage in parentheses.

^b^ Channel Islands/Isle of Man is contained in England outside London.

### 3.3 Distance Travelled Between Home and Specialist Care Centre

**FIGURE 4:**
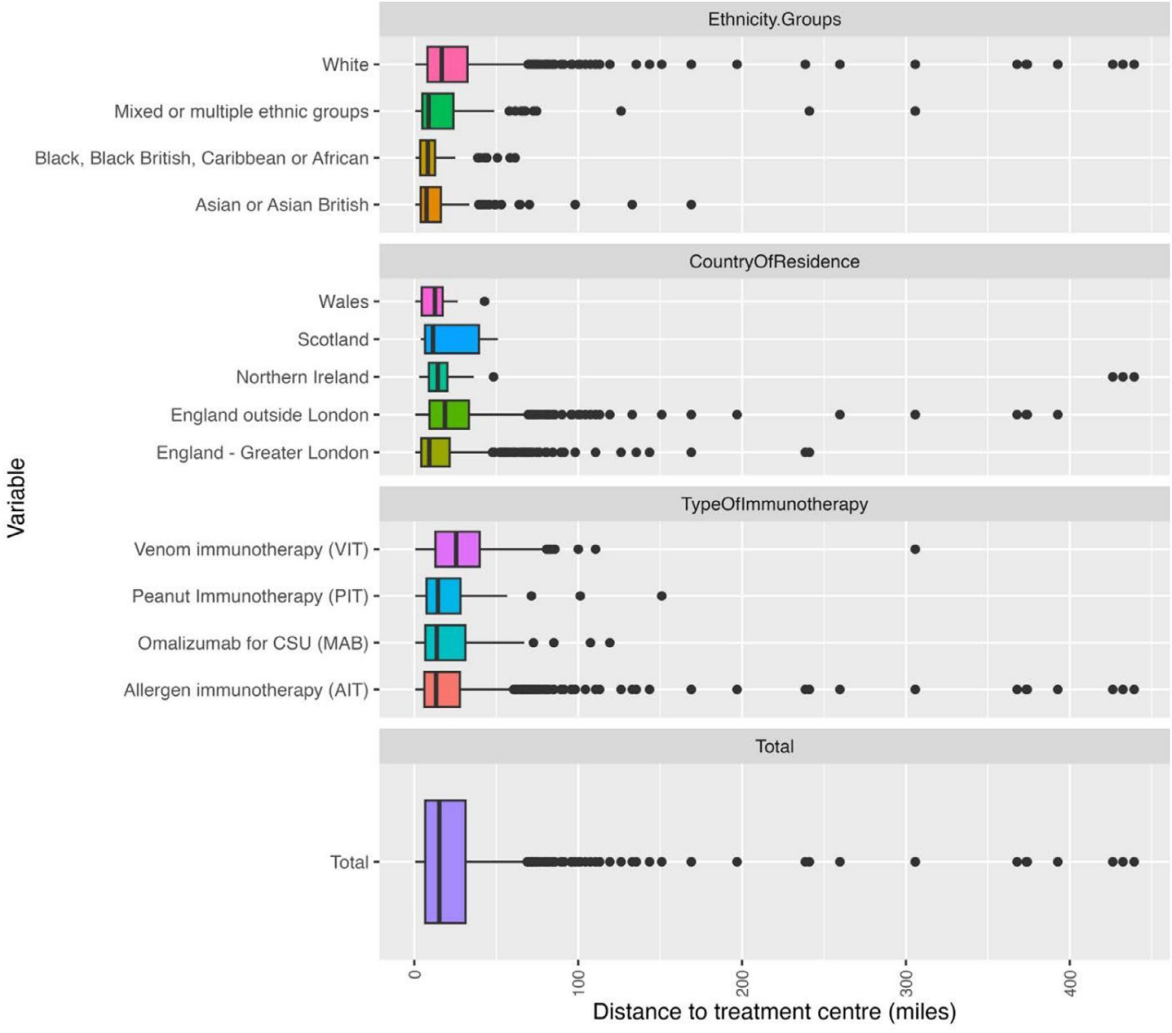
Charts showing the distance travelled to access services. The panels show box and whisker plots of the distance travelled between home postcode and specialist hospital in Miles. The uppermost panel shows distances according to ethnicity, followed by Country of residence, type of immunotherapy received, with the full registry distance travelled in the lowermost chart.

We calculated the distance travelled between home postcode and participant's specialist allergy centre (Figure 4). The median travel distance to access services was 15.2 miles. Travel was shortest in Greater London at a median distance of 9.1 miles (IQR 17.3), compared to 11.4 miles (IQR 32.9) in Scotland, 12.6 miles (IQR 12.8) in Wales, 14.3 miles (IQR 11.3) in Northern Ireland and 18.7 miles (IQR 24.0) in England outside London. We divided distances into terciles and assessed the empirical density to adjust the percentiles to represent three main clusters. The final terciles were defined as short travel distance under 9.2 miles (0%–35.0%), middle 9.3–19.9 miles (35.1%–60.0%) and long over 20 miles (60.1%–100%). Half of respondents (50.3%) travelled a short distance in London compared to 24.8% in England and 34.8% in the devolved nations. Participants were more likely to travel longer distances if they lived outside of London, in England (48.1%) and devolved nations (30.3%) compared with only 26.8% in London. Participants receiving venom immunotherapy travelled furthest, with a median distance travelled of 25.5 miles (IQR 27.0) as did those of white ethnicity, with a median of 16.8 miles (IQR 24.4) to receive care (Figure 4).

## 4 Discussion

The results suggest inequality in access to specialist allergy treatment in the UK. Not all forms of allergen immunotherapy and biological treatment are available throughout the UK. Access to care is related to geography and socioeconomic deprivation. Patients who were black or from mixed or minority ethnic backgrounds were less likely to be referred directly from primary care and were more likely to be referred from secondary or tertiary services. Difficulty in accessing treatment is also reflected by the distance travelled between home and hospital for specialist allergy care. Nearly 20 years after the House of Lords report on the unmet need of allergy sufferers in the UK, there are still inequalities in access to care [22].

### 4.1 Geographical Disparities

There is disparity in treatment around the UK. Using data from the 2021 census, the population of London is estimated at 7.5 million, compared to a combined population of 10.5 million in the devolved nations of Scotland, Wales and Northern Ireland [23]. Based on similar access to services in London, we would expect over 800 participants to have been registered from the devolved nations; instead, we had just 64. Similarly, using services in London as a benchmark, less than a third of the expected participants were accessing services from England outside of London in our sample (30.5%, 1193 of an estimated 3776). The London focus is related to the higher number of allergists in the capital [10].

Access to peanut oral immunotherapy was similarly underserved. Palforzia peanut OIT has not been approved for use in Scotland but has NICE approval for England and Wales. Approximately a quarter of a million (or 2% of 12.7 million) children in the UK have peanut allergy [24]. Using a conservative treatment rate of 2% of the 2% of children with peanut allergy, there should be 5000 children registered for PIT [25]. Instead, there were only 49 in the data set we used for the analysis, and of those, a third accessed care through private services rather than state‐funded health care. The proportion of peanut allergic children is also likely to be higher among minority ethnic groups. This is related to the increased risk of allergic disease and also due to barriers in access to dietary peanut allergy prevention in infancy [26, 27]. The almost complete absence of state‐funded PIT reported only increases the inequity for disadvantaged groups.

### 4.2 Socioeconomic Status and Ethnicity

Deprivation and ethnicity are also associated with access to services. The most affluent participants in our sample were three times as likely to access immunotherapy care within the NHS when compared to the most deprived. This is despite the NHS *Long Term Plan* to take action on those very inequalities and reduce unwarranted variation, although tackling the burden of severe allergic disease is not specifically mentioned in these goals [28]. The trend in treatment toward the more affluent was not seen in access to OMA for CSU. Access to OMA was evenly spread through socioeconomic status in keeping with the demographic characteristics of the disease [29].

There were differences in referral pathways between ethnic groups: those of black, black British and mixed or other minority ethnic groups were more likely to require referral from a secondary or tertiary care physician, whereas white and Asian or British Asian ethnic groups were more likely to be referred directly from their primary care providers. Ethnic minorities are more likely to experience significant allergic disease in the UK [30]. Similar inequalities in the provision of healthcare to ethnic minorities have been described in referral to mental health services and in routes to diagnosis for cancer [31, 32]. While language barriers and cultural differences in presentation may be part of the reason for these differences, we cannot exclude physician bias influencing referral [33]. The data suggest longer and more convoluted treatment pathways for patients who are at the greatest risk of severe disease, where prevention strategies and prompt referral for specialist care are most needed.

### 4.3 Cluster Effect Around Specialist Centres

There was a cluster effect of primary care referrals around specialist centres. It is likely that local primary care providers are aware of local specialist centres and make appropriate referrals, whereas those further away from centres do not know of their existence. This would suggest an education gap in primary care to know both how and where to refer for specialist allergy care. There is a need for targeted education, allergy‐aware primary care practices and regional referral networks, which could be fulfilled in part by patient support groups. The distance travelled for treatment is also important as most patients requiring allergen immunotherapy require multiple visits to a specialist centre. For some allergen immunotherapy, shared care models are available using sublingual immunotherapy that can be administered in any healthcare setting, but this would require appropriately trained physicians within primary care.

### 4.4 Limitations

Registry data have several limitations related to missing participants (unit nonresponse) and data points (item nonresponse). Participants need to give consent to be included in the registry, and there may be selection bias among those who enrol to participate. Several of the investigators have observed that BME participants were less likely to consent to have their data shared with the registry and that this was also true of the most affluent. However, the proportions of ethnic groups in the registry are broadly similar to those of the 2021 Census, where 9.3% are Asian/Asian British (c.f. 8.1% in the registry), 4.0% are black/black British (c.f. BRIT 3.3%) and 5.0% are mixed or other minority ethnic groups (c.f. BRIT 7.8%). There is no record kept of those who do not consent to participate, mainly because there is no ethical framework for holding such data. Not all centres contribute data to the registry; this may be more marked within the private sector. Use of BRIT is recommended as good practice by Improving Quality in Allergy Services (IQAS) the accreditation programme for allergy services managed by the Royal College of Physicians. Although there are allergy specialist centres for adults and children in all of the devolved nations, not all offer immunotherapy [7]. Based on analysis of services held by BSACI, it can be estimated that the registry covers two‐thirds of AIT centres at present, although the exact denominator is not known [34].

Missing data points are also common in clinical registries as, unlike clinical trials, they do not undergo the same level of data scrutiny. As this is a voluntary registry, we have attempted to keep data collection requirements to a minimum to reduce the burden on participants and practitioners [35].

Omalizumab was perhaps the least well reported of the treatment domains; this may be due to the use of OMA by dermatologists as well as allergists. There may also be competition from international registries like CURE [13], and some treatment centres may prefer to reserve their own data for their own research. There are advantages to being part of this project. Within BRIT, each centre and clinician has access to their own data for their own use, and by contributing data to the registry, they are also able to benchmark their practice against the registry as a whole using a real‐time dashboard. However, despite the limitations, this registry allows a detailed description of specialist allergy treatments in the UK.

### 4.5 Next Steps

All centres practising allergen immunotherapy and biologic support for Chronic Spontaneous Urticaria in the UK should be encouraged to engage with the registry for the benefit of their patients and services. Commissioners should consider funding based upon contributions to this national dataset. To improve referrals, there is a need for increased visibility of allergy centres to primary medical care with clear commissioning pathways in place.

Children are well represented among BRIT participants. This reflects the expansion of the provision of paediatric allergy care. There is a need to increase adult allergy training posts across the UK to lead the development of the specialty [1, 10, 35]. Ethnic minorities in the UK are more likely to have significant allergic disease [30]. The need to improve engagement is multifactorial and has been discussed recently, highlighting the issues associated with urban deprivation in the United States of America [36]. There is a need to work with minority ethnic communities to understand the barriers to accessing specialist services and develop appropriate interventions [37].

## 5 Conclusion

This is the largest and most comprehensive data on the use of allergen immunotherapy in the UK. There are disparities in the provision of treatment around the UK and particularly the absence of registry participants from the devolved nations. Access to NHS care is influenced by geography, socioeconomic deprivation and ethnicity. There were differences in referral pathways to specialist allergy services based upon ethnicity: with black people, those of mixed ethnicity and other minority ethnic group participants less likely to be referred directly from primary care. The BSACI Registry for Immunotherapy has highlighted inequity in access to specialist allergy services in the UK.

## Author Contributions

M.E.‐L. designed the study, methodology, and analysis plan with V.B., and with S.S. was responsible manuscript drafting. L.P.V. and O.M. undertook statistical analysis. M.S. provided registry database oversight. Other authors contributed to the study's conceptual framework and provided clinical insights. They reviewed and edited the manuscript for intellectual content. The BRIT Practitioners Group oversaw participant recruitment and clinical data collection, and provided critical revisions to the manuscript. All authors and collaborators reviewed and approved the final manuscript.

## Ethics Statement

BRIT is a research database approved by the West of Scotland Research Ethics Committee 4 (IRAS Number 249481).

## Conflicts of Interest

M.E.‐L. has received advisory board honoraria and speaker fees from Allergy Therapeutics and DBV Technologies. He is supported by Southampton NIHR Biomedical Research Centre. T.D. reports ALK Albelo provided travel and conference registration fees to members of his department (2023). L.M. has previous (2020) speaker fees, Advisory Boards and Commercial Clinical Trial Research Grants from Danone Nutricia and Regeneron unrelated to this work and submission. Other authors declare no conflicts of interest.

## Data Availability

The data that support the findings of this study are available from the British Society for Allergy & Clinical Immunology. Restrictions apply to the availability of these data, which were used under licence for this study. Data are available on application to BSACI with the review and permission of the Registry Steering Committee.

## Appendix 1

Hana Alachkar^12^; Saul Berkovitz^27^; Malini Bhole^26^; Thippeswamy Billahallli^7^; Helen A Brough^4,10^; Natalia Cartledge^23^; José Maia e Costa^15,19^; Beverley Fish^8^; Minal Gandhi^22^; Pavels Gordins^8^; Elizabeth Griffiths^28^; Sophie Hadfield^14^; Haseena Jearally^1^; Anjli Jethwa^29^; Leah Jones^20^; Sonia Lal^25^; Claire Leck^7^; Sian Ludman^21^; Susana Marinho^13^; Joanne McCullough^14^; Anne McDonnell^14^; Hayley Meers^11^; Phoebe Moulsdale^31^; Lizanne Noronha^10^; Nandinee Patel^9^; Julie Pentland^14^; Raul Scott Pereira^6^; Graham Roberts^28^; Jennifer Rowley^5^; Anna Rucker^23^; Michael Rudenko^27^; Chris Rutkowski^4^; Penny Salt^27^; Aida Semic‐Jusufagic^11^; Punit Shah^3^; Sarah Sholtysek^8^; Adrian Sie^18^; James Stewart^17^; Gary Stiefel^30^; Iason Thomas^13^; David Tuthill^2^; Gillian Vance^14^; Shaun Walter^10^; Patrick Yong^16^; Natasha Zurick^24^.

1. Barts Health NHS Trust

2. Cardiff and Vale University Health Board

3. County Durham and Darlington NHS Foundation Trust

4. Guy's and St Thomas' NHS Foundation Trust

5. Hampshire Hospitals NHS Foundation Trust Basingstoke

6. Harley Street Clinic, HCA Healthcare

7. Homerton Healthcare NHS Foundation Trust

8. Hull University Teaching Hospitals NHS Trust

9. Imperial College Healthcare NHS Trust

10. King's College Hospital NHS Foundation Trust

11. Leeds Teaching Hospitals NHS Trust

12. Liverpool University Hospitals Foundation Trust

13. Manchester University NHS Foundation Trust

14. Newcastle Hospitals NHS Foundation Trust

15. New Foscote Hospital

16. NHS Frimley Health Foundation Trust

17. NHS Grampian

18. NHS Lanarkshire

19. Nuffield Hospital, Warwickshire

20. Oxford University Hospitals NHS Foundation Trust

21. Royal Devon University Healthcare NHS foundation trust

22. Royal Free London NHS Foundation Trust

23. Royal Surrey NHS Foundation Trust

24. Royal United Hospitals Bath NHS Foundation Trust

25. Sandwell and West Birmingham Hospitals NHS Trust

26. The Dudley Group NHS Foundation Trust

27. University College London Hospitals NHS Foundation Trust

28. University Hospital Southampton NHS Foundation Trust

29. University Hospitals Coventry and Warwickshire NHS Trust

30. University Hospitals of Leicester NHS Trust

31. Worcestershire Acute Hospitals NHS Trust

The BRIT committee would like to acknowledge support and assistance from all registered BRIT users. Additional acknowledgments: Dendrite Team (Julie Dawson, Dave Kilburn, Chris Rixon, Andy Smallman, and Peter Walton). BSACI CEO Fiona Rayner.
